# Dose-related effects of extracorporeal shock waves on orthodontic tooth movement in rabbits

**DOI:** 10.1038/s41598-021-82997-5

**Published:** 2021-02-09

**Authors:** Onur Demir, Nursel Arici

**Affiliations:** Department of Orthodontics, Faculty of Dentistry, Ondokuz Mayıs University, Atakum, Samsun, Turkey

**Keywords:** Biomarkers, Medical research

## Abstract

The purpose of this animal study is to investigate the quantitative effects of extracorporeal shock waves applied at two different impulses and with two different applicators on orthodontic tooth movement. Thirty-five New Zealand rabbits were randomly divided into five groups (n = 7): the four experimental extracorporeal shock wave groups—focused/500 impulses, focused/1000 impulses, unfocused/500 impulses, and unfocused/1000 impulses—and the control group. Orthodontic tooth movement was achieved by application of reciprocal force between two maxillary incisors. In the experimental groups, animals received 500 or 1000 impulses of extracorporeal shock waves at 0.19 mJ/mm^2^ with focused or unfocused applicators depending on the group to which they belonged. These experiments were conducted on days 0, 7, and 14. Orthodontic tooth movement was measured with 0.01 mm accuracy at one-week intervals. On days 7 and 21, the bone-specific alkaline phosphatase levels were measured from blood samples. After 21 days, the animals were sacrificed and the area between the two maxillary incisors was stereologically examined. Orthodontic tooth movement in the focused/500 impulses and focused/1000 impulses groups was significantly increased compared to the control group. A significant difference in bone-specific alkaline phosphatase levels between the unfocused/500 impulses and control groups was found at 21st day. Stereological analysis showed that there were significant increases of the formation of new bone, connective tissue, and vessels in the experimental groups. The application of extracorporeal shock waves, especially with a focused applicator, could accelerate orthodontic tooth movement.

## Introduction

Orthodontic treatment aims to improve individuals’ quality of life by correcting malocclused teeth. Achieving tooth movement during this treatment is a complex and prolonged inflammatory process that involves simultaneous bone resorption and apposition. The main complaint of patients seeking orthodontic treatment is the length of the treatment process^1^. In addition, prolonged application of force during treatment may be associated with certain risks, such as caries, pain, increased mobility, root absorption, periodontal diseases, and decreased patient cooperation^2,3^. Due to the growing demand for orthodontic treatment, despite improvements in orthodontic materials and the efficiency of mechanical forces, metabolic limits have led researchers to look for different ways to accelerate tooth movement. For example, some researchers have tried to apply chemical agents, electric currents, and electric impulsed electromagnetic fields^4–6^. Recent studies have reported that local application of lasers, vibration, or corticotomy can accelerate orthodontic tooth movement (OTM)^7–9^. Extracorporeal shock waves (ESWs) have also been identified as a valuable method for accelerating OTM^10,11^. ESWs are a non-invasive, mechanical form of high-level sound wave treatment with different magnitudes of stimuli. This type of treatment promotes regenerative abilities of tissues without any adverse effects in the oral cavity and could increase osteoblastic and fibroblastic activation in the bone and connective tissue^12–16^. Based on the assumption that these shock waves may have an accelerating effect on tooth movement, some studies have been conducted on the use of ESWs in orthodontics^10,11,13–15^. However, the energy flow intensity, pulse number, frequency, and pressure values of the shock waves that will produce the optimum biological effects during OTM remain unclear. Moreover, there is no study comparing the effects of focused and unfocused applicators on OTM. Therefore, the aim of this controlled animal study is to investigate the effects of weekly applications of ESWs with two different impulse values and with focused and unfocused applicators. The null hypothesis states that changing the frequency of application time, impulse value, and focusing parameters of ESW does not affect OTM.

## Material and methods

The sample size of this animal study was calculated in accordance with a study investigated OTM under orthodontic force loading in a rabbit model^17^. The number of animals was set at six per group based on the effect size (1.676) at an alpha level of 0.05 and a power of 80% (determined using the statistical software G*Power 3.1.9.2., https://gpower.software.informer.com/download/). Due to the potential problems that may be encountered in this study, the sample size was increased by 1 per group and 35 (5 × 7) in total.

### Animals

All treatments in this experimental study were performed on 35 female New Zealand albino rabbits. All procedures were approved and regularly controlled by the Animal Ethics Committee of Ondokuz Mayıs University (No: OMU HADYEK 2012/30), and all experiments were performed in accordance with the guidelines and regulations of this committee. All the procedures were also carried out in full accordance with the ARRIVE guidelines and adequate care was taken to minimize pain and discomfort for animals.

During the experimental period, rabbits were allowed free access to water and a standard pelleted food diet. The temperature was 21 ± 2 °C, and the humidity was 50 ± 10%. All animals received the same 12/12-h cycle of light/dark environmental light. The rabbits were randomly divided into five groups (n = 7): four ESW experiments—focused/500 impulses (F1), focused/1000 impulses (F2), unfocused/500 impulses (U1), and unfocused/1000 impulses (U2)—and the control (C) group.

### Experimental design

**Figure 1:**
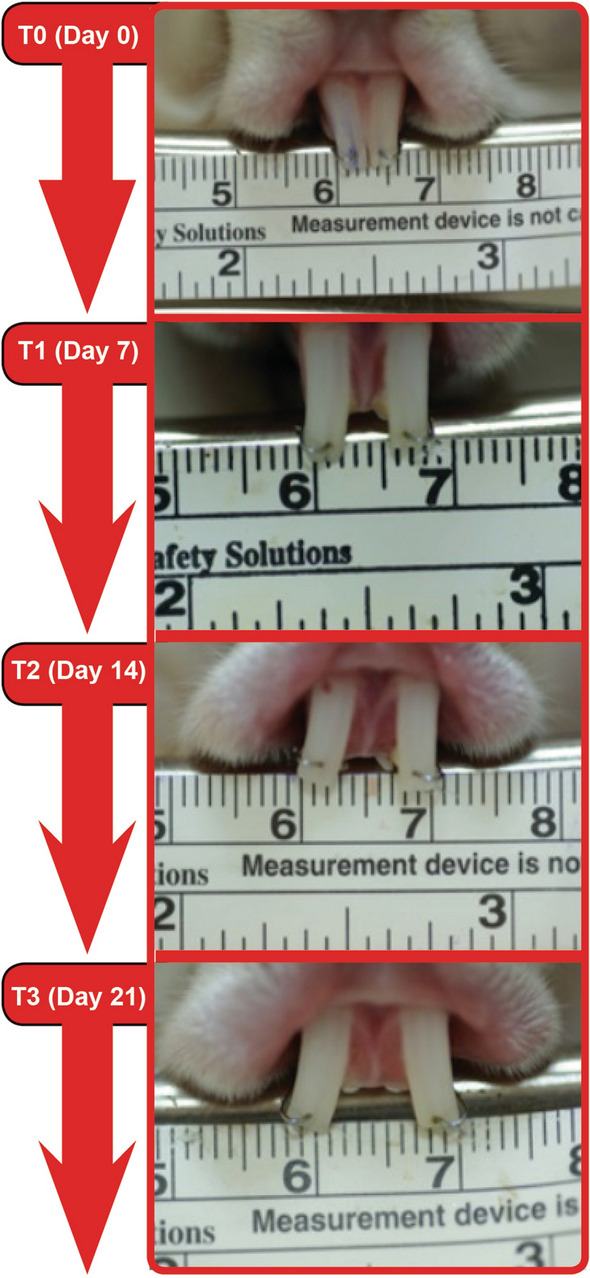
Intra-oral pictures taken of the maxillary incisors of a rabbit in the F1 group during the experiment.

To produce standard orthodontic forces, springs constructed from 0.4 mm (0.016-inch) round stainless steel wires were used^18^. The arms of the springs were 13 mm long, and the angle between the two arms was set at 70°. In all groups, the rabbits were mildly anesthetized with an intramuscular injection of 40 mg/kg ketamine and 5 mg/kg xylazine. Then, they were placed in a special fixing device before spring installation. The upper first incisors were drilled in the vestibulo-palatal direction using a bur under physiological saline cooling at a distance of 1.5 mm from the incisal edges. The ends of the spring arms were placed in these holes, and a total of 60 g of reciprocal force was applied. In other words, 30 g of distalization force was applied to each of the maxillary incisors. After application of force, the conditions of the rabbits and springs were checked by the same investigator every 24 h for 21 days. Standard photographs were taken on days 0 (T0), 7 (T1), 14 (T2), and 21 (T3) using a special apparatus to record dental movement (Fig. 1). These photos were transferred to a digital image program (Image J, Maryland, USA) and calibrated using the ruler in the photograph. Then, the adjacent surfaces of the two maxillary incisors were digitally marked at the level of the alveolar crest, and the distance between them was measured on a line drawn parallel to the alveolar crest.

### ESW applications

**Figure 2:**
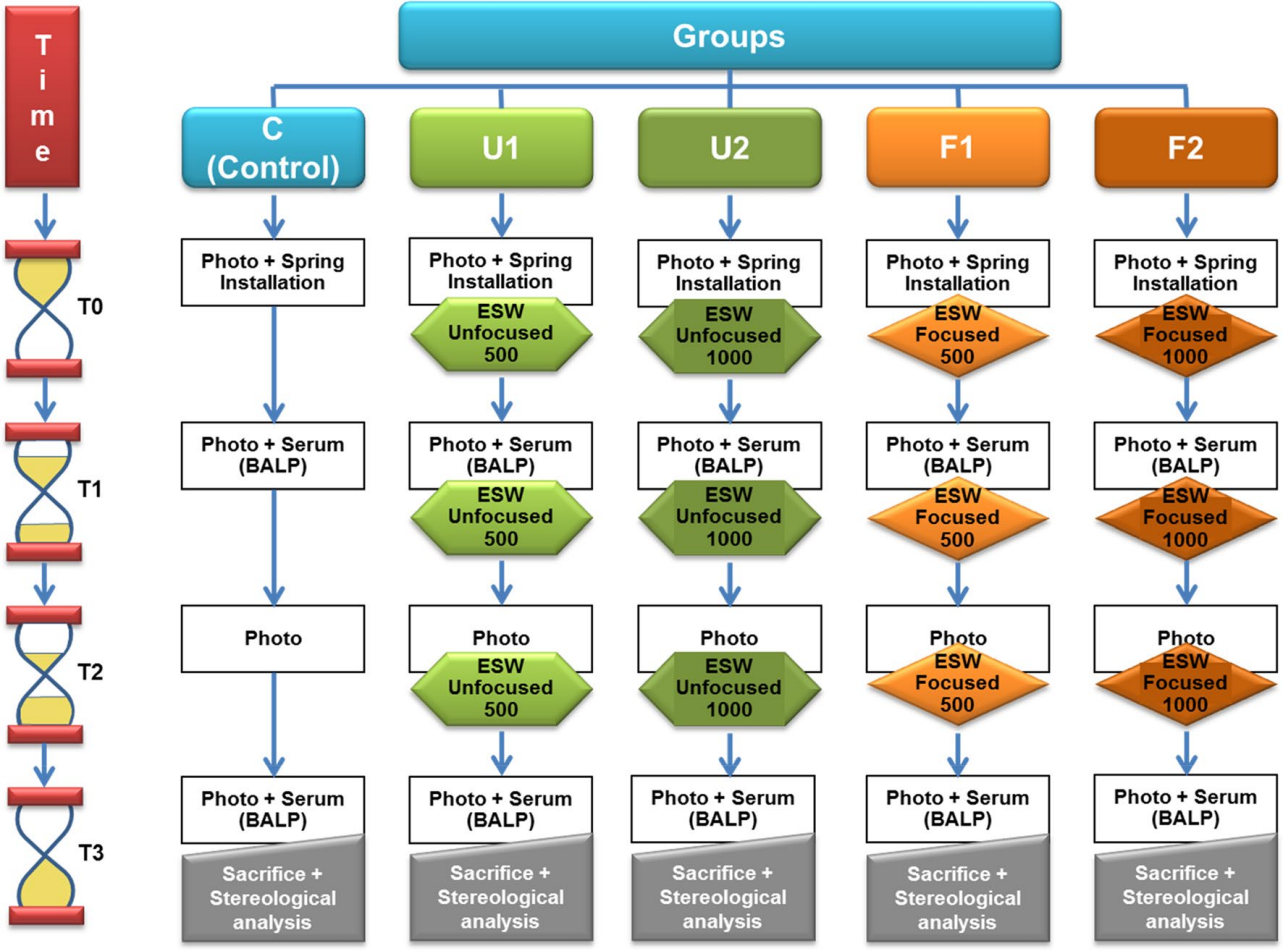
Flowchart of the study.

ESW was applied to rabbits with a medical ESW device (MTS Orthogold 100, Germany). The rabbits were anesthetized as described above before each ESW application. Two applicators, one focused and one unfocused, were used (focused OE50 and unfocused OP155, Orthogold 100, MTS, Konstanz, Germany). In the ESW groups, a single shock wave treatment of 500 or 1000 focused or unfocused impulses was used at an energy flux density 0.19 mj/mm^2^ with a pulse rate of 5 pulses per second. Half of the impulses were applied at right angles to the right maxilla, and half were applied to the left maxilla. Before ESW, an ultrasound transmission gel was applied to the target areas. This protocol was repeated at the T0, T1, and T2 time points for each animal in the ESW groups. ESWs were not applied to the animals in the control (C) group, but records were taken in the same way. The flowchart in Fig. 2 shows the application times and properties of the ESW used in this study, as well as all other records taken.

### Biochemical examination

After routine anesthetic procedures, between 9:00 am and 11:00 am at the T1 and T3 time points, 6–8 cc of blood was collected from the rabbits’ ear veins with blue intricate for serum samples. Serum samples were taken before ESW application at the T1 time point (Fig. 2). Following a centrifugal process, samples kept in 1 cc Eppendorf tubes were stored at − 80 °C for biochemical analysis. The level of bone-specific alkaline phosphatase (BALP), which is an important bone turnover enzyme^19^, was examined using the enzyme-linked immunosorbent assay (ELISA) method using a rabbit-specific BALP ELISA kit (Rabbit-BALP, QAYEE, China). The microplate was analysed with an absorbance reader (Sunrise™, Tecan, Switzerland).

### Stereological analysis

At the end of the 21 days (T3), 30 rabbits were sacrificed with high-dose sodium pentobarbital. First, the external soft tissues were removed, and the maxilla and teeth were resected. The tissues were decalcified in 5% formic acid for 21 days and then fixed in 10% formaldehyde. After routine histological procedures, 7-µm-thick serial sections from the area between the two maxillary incisors were taken using a rotary microtome (Leica RM 2135RT; Leica Instruments, Nussloch, Germany). One out of every 100 sections was chosen according to systematic random sampling strategies and stained with hematoxylin–eosin (HE). Then samples were photographed by using light microscopy (Leica M 4000 B, Germany) with a colour digital camera (Microbrightfield, Williston, VT) in a stereological analysis system (Stereoinvestigator 9.0, Microbrightfield, USA).

The total volumes of new bone, connective tissue, and new capillaries were estimated using the Cavalieri method^20^ on light microscopy images in a computer environment. When determining area, point-counting grids (each grid point represents 5000 µm^2^) were applied to images. After performing a pilot study, point density was determined based on the acceptable coefficient of error (CE), which was less than 0.05 for each sample. Regarding the coefficient of variation (CV) values of each group, six samples were found to be enough to represent the relevant group, stereologically. For Cavalieri volume estimation, the following formula was used:

$${\text{Volume}} = {\text{t}} \times {\text{a}}/{\text{p}} \times \Sigma {\text{p}},$$

where t is section thickness, a/p is the area representing each point on the point-counting grid, and Σp is the total number of points in the area between the two maxillary incisors.

### Statistical analysis

The study was planned as a parallel-group, blind, randomized, controlled, experimental animal study. Randomization was performed by computer-generated random codes. Laboratory procedures, application of the orthodontic apparatus and ESWs, capture of intra-oral photos, and collection of blood samples were performed by a researcher who knew the groups to which the subjects belonged (O.D.). OTM was measured by the other researcher (N.A.) based on the photographs, matching of the BALP levels, and stereological volume measurements from the laboratories. This researcher did not know the groups to which the subjects belonged. A biostatistician blind with respect to the experiment design performed the analysis.

The data were analysed using a statistical software package program (SPSS, v. 21; IBM New York, USA). Since the data were normally distributed in all groups according to the Shapiro–Wilk normality test, a repeated-measures analysis of variance (multiple ANOVA) was performed for the samples to compare the OTM within the groups at the three time points. In addition, to determine the effects of ESWs on the OTM, BALP levels, and stereological volume estimation (SVE) values of the groups, a one-way ANOVA was performed. This was followed by Tukey’s honestly significant difference (HSD) test for multiple comparisons of means, which had a p-value of < 0.05, to determine differences among the different groups.

### Ethics approval

The study was approved by the Animal Ethics Committee of Ondokuz Mayıs University (No: OMU HADYEK 2012/30).

## Results

The means, standard deviations of the OTM, BALP levels, and SVE values for the groups are listed in Table 1.

**Table 1:** Summary of OTM, BALP, and SVE results.

	Time	n	Unit	Group					p
				C	U1	U2	F1	F2	
				Mean (sd)	Mean (sd)	Mean (sd)	Mean (sd)	Mean (sd)	
OTM	T1	7	mm	2.53 (± 0.300)	2.87 (± 0.314)	2.94 (± 0.768)	3.21 (± 0.303)	3.66 (± 0.619)	**
	T2	7	mm	3.50 (± 0.580)	3.95 (± 0.887)	4.09 (± 1.236)	4.56 (± 0.445)	4.45 (± 0.715)	NS
	T3	7	mm	4.54 (± 0.288)	5.02 (± 1.061)	5.23 (± 0.894)	5.37 (± 0.403)	5.77 (± 0.583)	*
BALP	T1	7	pg/ml	86.60 (± 23.32)	78.91 (± 8.38)	68.26 (± 13.76)	70.70 (± 13.81)	84.62 (± 14.36)	NS
	T3	7	pg/ml	86.71 (± 17.66)	64.51 (± 13.11)	89.42 (± 4.79)	78.66 (± 12.23)	97.80 (± 16.40)	**
**Stereology**									
N. Bone	T3	6	mm^3^	2.71 (± 0.08)	2.75 (± 0.05)	2.71 (± 0.04)	2.84 (± 0.03)	2.89 (± 0.11)	***
N. Con Tissue	T3	6	mm^3^	0.50 (± 0.06)	0.64 (± 0.04)	0.67 (± 0.11)	0.68 (± 0.05)	0.75 (± 0.08)	***
N. Vessels	T3	6	mm^3^	0.25 (± 0.01)	0.28 (± 0.01)	0.28 (± 0.02)	0.34 (± 0.01)	0.38 (± 0.03)	***

U1 = unfocused/500 impulses, U2 = unfocused/1000 impulses, F1 = focused/500 impulses, F2 = focused/1000 impulses, and C = control groups.

T1 = 7th day, T2 = 14th day, and T3 = 21st day.

*p < 0.05, **p < 0.01, ***p < 0.001.

### OTM

**Figure 3:**
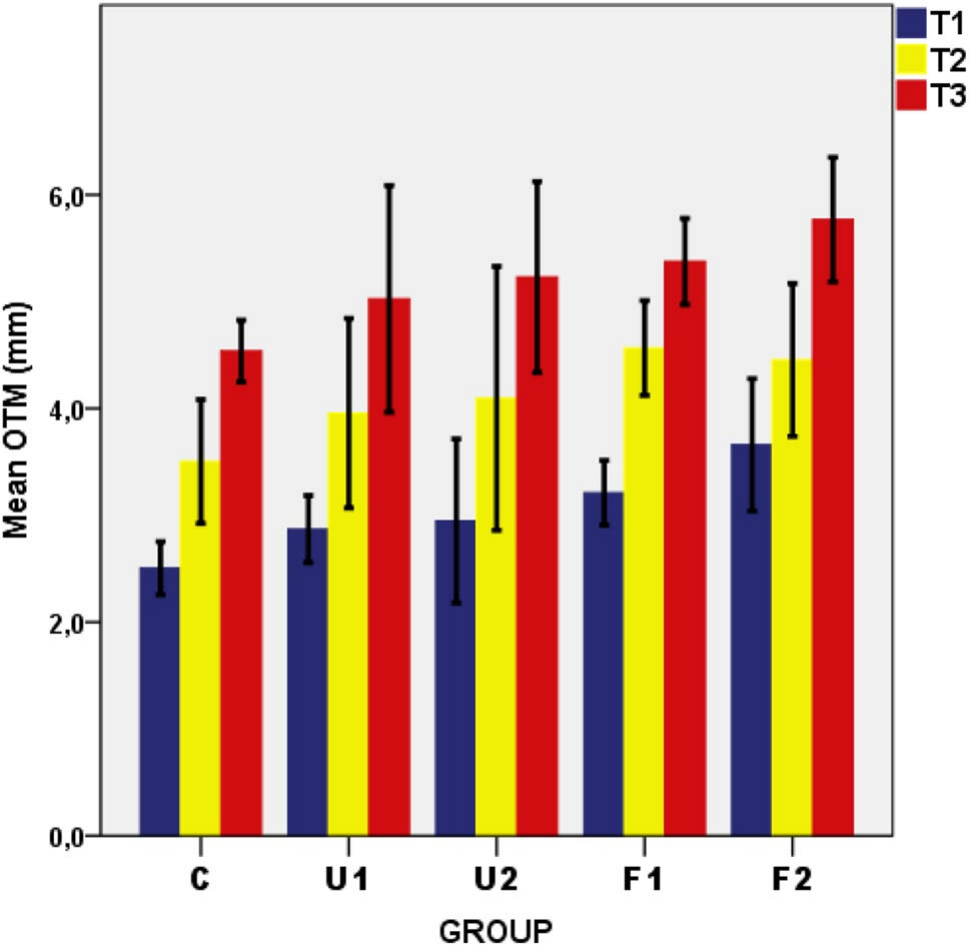
Graphical representation of the mean OTM values and standard deviations of the groups in three time intervals.

The lowest mean OTM value (2.53 ± 0.30) was found for the control group at T1, followed by the U1 group (2.87 ± 0.31). The mean OTM values of all groups at T1 were approximately half those of their counterparts at T3. The F2 group had the highest mean OTM value (m = 5.77 ± 0.58) of all groups at T3 (Table 1 and Fig. 3).

A repeated-measurements ANOVA was used to test the hypothesis that there was no significant difference between the OTM values of the groups across the three time points. It showed that the effect of time was significant (F = 248.79, p = 0.000) at the 95% confidence level. In other words, the results indicate a significant time effect for OTM within the groups on a weekly time scale. Comparison between the groups was performed using a one-way ANOVA, which showed significant differences between the groups (Table 1). The groupings of these differences obtained from Tukey’s HSD multiple-range test indicated that the C (2.53 ± 0.30) and U1 (2.87 ± 0.31) groups had significantly lower mean OTMs than the F2 group (3.66 ± 0.61) at T1. There were no significant differences among the groups at T2. At T3, the F2 group (5.77 ± 0.58) showed significantly higher OTM than the C group (4.54 ± 0.28) (Table 2).

**Table 2:** Statistically significant differences among the five groups using Tukey’s HSD multiple-range test (filled squares show significantly different pairs at the 95% confidence level, * p < 0.05, ** p < 0.01, *** p < 0.001).

Measurement	Time	Groups				
			U1	U2	F1	F2
**OTM**		C				**
	T1		U1			*
				U2		
					F1	
			U1	U2	F1	F2
		C				*
	T3		U1			
				U2		
					F1	
**BALP**			U1	U2	F1	F2
		C	*			
	T3		U1	*		**
				U2		
					F1	
**SVE** New Bone			U1	U2	F1	F2
		C			*	**
	T3		U1			*
				U2	*	**
					F1	
New Con. Tissue			U1	U2	F1	F2
		C	*	**	**	***
	T3		U1			
				U2		
					F1	
New Vessels			U1	U2	F1	F2
		C			***	***
	T3		U1		***	***
				U2	**	***
					F1	**

U1 = unfocused/500 impulses, U2 = unfocused/1000 impulses, F1 = focused/500 impulses, F2 = focused/1000 impulses, and C = control groups.

T1 = 7th day, T2 = 14th day, and T3 = 21st day.

### Biochemical findings

**Figure 4:**
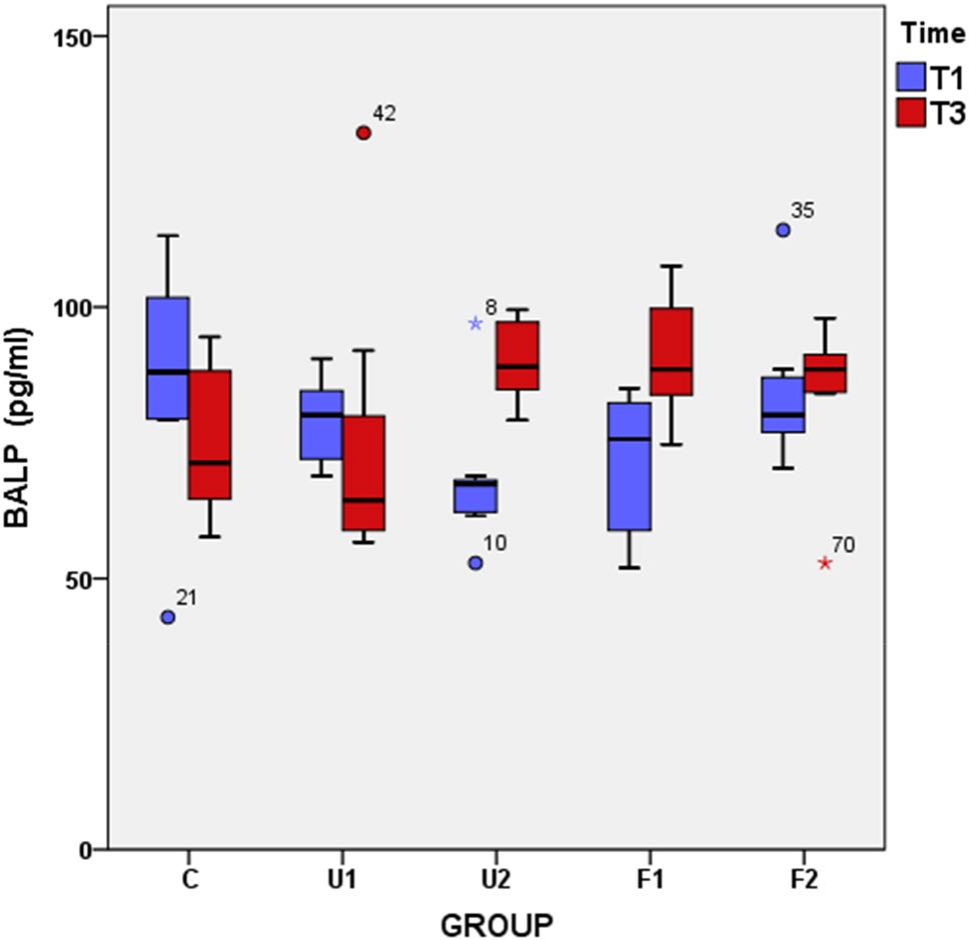
Graphical presentation of the changes in the serum bone-specific alkaline phosphatase (BALP) level from time T1 (day 7) to T3 (day 21) in groups.

Pairwise comparisons within the groups, which were performed with a paired-samples t-test, revealed that the U2, F1, and F2 groups at the T1 time showed significantly higher BALP values than their counterparts at T3 (p < 0.05). There was no significant difference between the BALP values of the C group at T1 and T3 (p = 0.98). However, the mean BALP level of the U1 group decreased from T1 to T3, and this decrease was significant (p < 0.05). Comparison between the groups at the same time points was performed using a one-way ANOVA, and significant differences were found among the groups at T3 (Table 1 and Fig. 4). The grouping of these differences indicated that the U1 group had a significantly lower BALP level than the C, U2, and F2 groups at T3 (Table 2).

### Volumetric assessment

**Figure 5:**
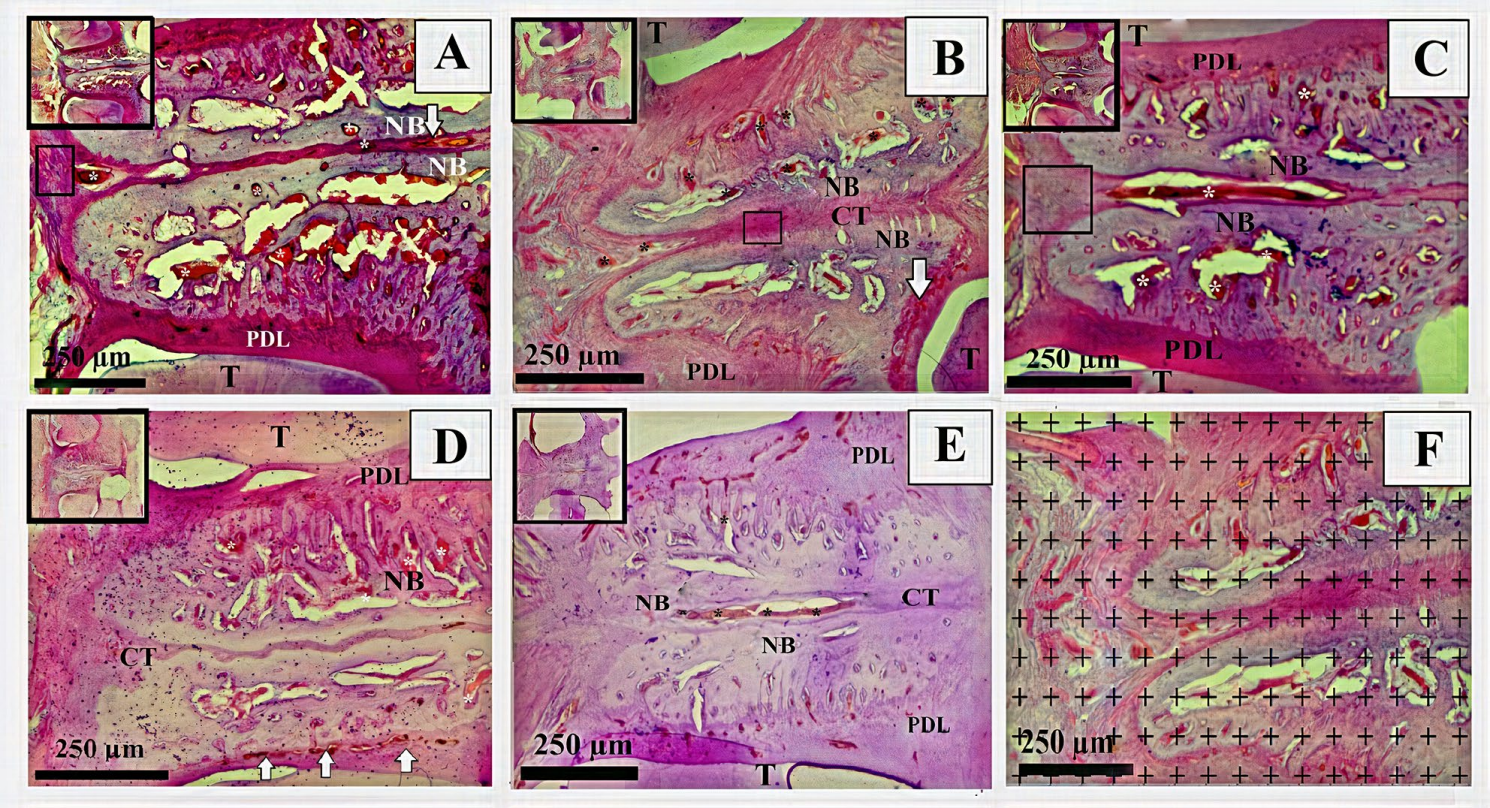
Histological representative light microscopic images of the groups (**A**) F2, (**B**) F1, (**C**) U2, (**D**) U1, (**E**): C and the point counting grid for Cavalieri volume estimation (**F**). NB: new bone; white arrows: new capillaries; *CT* connective tissue; small squares: infiltration areas; *PDL* periodontal ligament; T: tooth; larger squares: panoramic view of distracted areas (hematoxylin & eosin staining).

**Figure 6:**
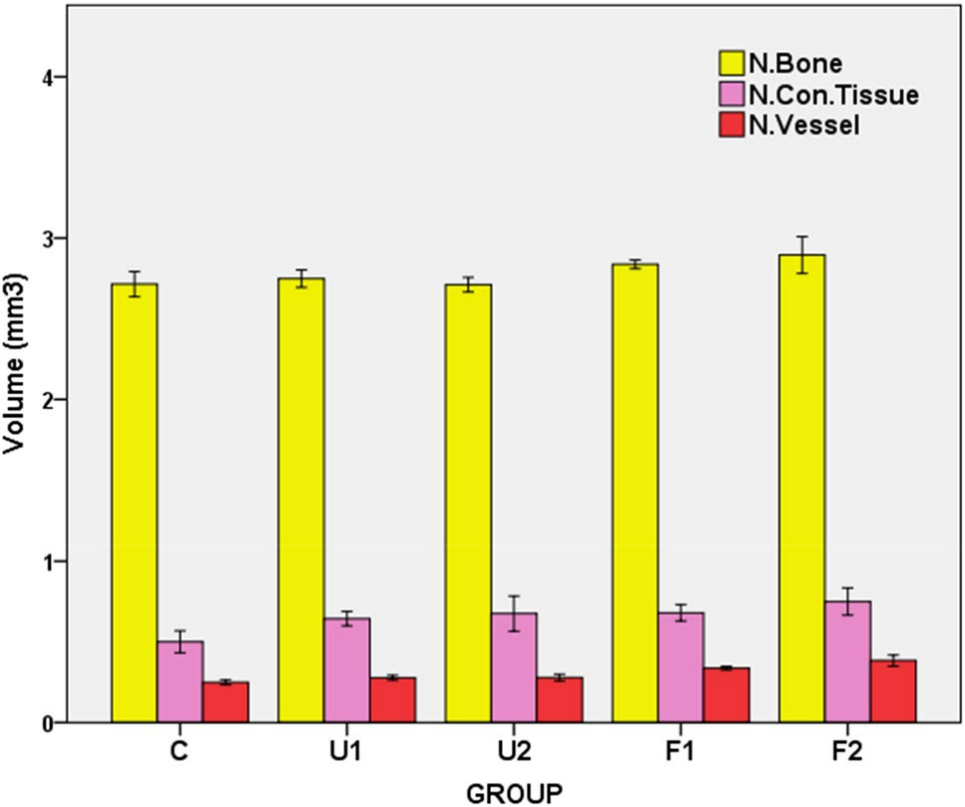
The mean and standard deviation values of new bone, new connective tissue and new vessel formation in groups.

Based on volume measurements, the F2 group showed the highest mean values for new bone (2.89 ± 0.11), connective tissue (0.75 ± 0.08), and vascular formation (0.38 ± 0.03), while the C group had the lowest values for all of these measurements (2.71 ± 0.08, 0.50 ± 0.06, and 0.25 ± 0.01, respectively) (Table 1 and Figs. 5 and 6). A post-hoc Tukey’s HSD multiple-range test indicated that, in terms of newly formed bone volume, the F1 and F2 groups had significantly higher values than the C group (p < 0.05 and p < 0.01, respectively) and the U2 group (p < 0.05 and p < 0.01, respectively) (Table 2). In addition, the F2 group had a significantly higher mean value than the U1 group (p < 0.05).

When groups were compared in terms of newly formed connective tissue volume, group C had significantly lower mean values than all experimental ESW groups (Table 2). However, there was no significant difference between the experimental ESW groups (Table 2). Additionally, significant differences were found in the newly formed capillary volumes between the groups. The C group had a significantly lower mean value than the F1 and F2 groups (p < 0.001), and the F1 and F2 groups were statistically significantly different from each other (p < 0.01) and from all the other groups (Table 2 and Fig. 6).

## Discussion

In recent years, innovative approaches have been developed to shorten long treatment processes, which are considered the biggest disadvantage of orthodontic treatments. Although ESW application is one of these approaches, studies focusing on it are limited^10,11,14–16,21^, and not all of the variables have been investigated to determine their effect on OTM. Thus, in this study, the effects of different components of ESW application, such as impulse number and application area for OTM, were investigated, and the biochemical and stereological findings as well as OTM were evaluated.

Although data from animal studies are sometimes difficult to extrapolate to humans, animal models for OTM are very useful in obtaining information about the biological response to orthodontic forces. Rabbits used for orthodontic research^17,18,22–24^ were preferred in this study because of their physical size was suitable for ESW applicators, the ease of serum collection, the ease of care throughout the 21-day working period, their short life span, the presence of teeth suitable for orthodontic mechanics, and faster bone turnover^18,23,24^. In addition, they reach skeletal maturity shortly after sexual maturity at six months^25^. The incisors and/or molars of rabbits were used to investigate OTM^18,22–24^. In this study, maxillary incisors were preferred in order to keep the application area of the ESW treatment away from the brain, create faster tooth movements, allow the alveolar bone to respond to extracellular stimuli faster, and apply the mechanics easily. However, rodents possess long-crowned, continuously growing and open-rooted teeth^26^. Maxillary incisors of rabbits can grow up to approximately 1.9 mm per week depending on the type of food they are fed^27^. This physiological eruption of the incisors can cause the level and amount of force applied by the spring to change. Fortunately, this continuous growth is not constant and generally compensates for the wear of the respective tooth^28^. In this study the animals received a constant diet during 21 days of experiment. Therefore, it can be said that the amount of physiological tooth eruption that will occur during the experiment is not long enough to disturb the occlusal balance and affect the results obtained in this study.

In experimental studies, 28–170 g of force was applied by springs with different designs in order to move the rabbits' incisors^18,22–24^. In the present study, 60 g of reciprocal force was applied between two maxillary incisors via a spring, as described elsewhere^18^, and no side effects due to springs, such as food retention, tissue damage, or opening of the mid-palatal suture, were observed.

Although some previous experimental animal studies have taken a few minutes or a few months, it seems that most experimental studies examining orthodontic tooth movement in rabbits last 20–21 days^29^. It has been reported that when 60 g of force is applied, the cycle of tooth movement is seven days in young rats^30^ and the most active bone reshaping response was observed on the seventh day after force application^31^. Considering this cycle, ESW was applied at seven-day intervals (T0, T1, and T2), and a 21-day experiment period was chosen for this study. The results of some studies revealed that EWS application in orthodontic treatment does not have an invasive effect on dental and surrounding tissues^10,11,15^. These findings were supported by the first clinical study in which a single dose of ESW was applied to humans during OTM^11^. However, it was also stated that ESW applications with multiple or high-energy flow concentrations may have different effects on these tissues^11^. Additionally, in an experimental rat study, a single application of ESWT of 500 focused impulses was applied at an energy flux density of 0.1 mj/mm^2^ with a pulse rate of 5 pulses per second at the time that tooth movement was initiated. This increased osteoblast and osteoclast activities and imbalanced bone remodelling, resulting in impeded tooth movement^21^. In experimental animal studies evaluating the effects of ESW on dentofacial tissues, 500 or 1000 focused or unfocused extracorporeal shock waves were usually applied at an energy flux density of 0.10—0.25 mJ/mm^2^ with 5 pulses per second^10,16,21^. Therefore, 500 or 1000 focused or unfocused extracorporeal shock waves at an energy flux density of 0.19 mJ/mm^2^ at 5 pulses per second were used in this study. The present study is also the first to evaluate the effect of weekly ESW applications at different doses on OTM.

ESW application is known to have different effects on organisms, such as extracellular cavitations, ionized molecules, changes in cell membrane polarization and permeability, formation of radicals, and microcrack formation^32^. The reaction caused by orthodontic force with mechanotransduction in the periodontium activates similar systems, physiologically and/or pathologically^33^. Due to the presence of similar cellular responses in both applications, it is possible to expect an increase in OTM as well as a cumulative effect when both applications are combined. Indeed, in the present study, the mean OTM values of all experimental groups are higher than those of the C group at all time periods. These results are consistent with the results of other study that applied ESW during OTM^11^. However, in the present study, only the difference between the F2 and C groups was statistically significant at T1 and T3. First, we thought that this might be related to the number of subjects in the groups. In a rat study, the researchers reported that osteoclasts originating from the periodontium functioned at the beginning of OTM, and when orthodontic force was applied for a long time, osteoclasts originating from far hematopoietic organs’ cells exhibited resorption functions^34^. However, another study reported that osteoclasts do not originate from the hematopoietic organs or periodontium, but from the bone marrow^35^. Therefore, it was suggested that alveolar bone, instead of periodontal ligaments or gingiva, should be targeted to achieve a biostimulant effect^35^. The results of these studies might explain the lack of a significant difference between the mean OTM values of the C group and experimental groups at T2 in the present study. The increase in these measurements after the second week may be due to the biostimulative effect of ESW application on the alveolar bone marrow, which is the source of osteoclast internal migration in the maxillary region.

Alkaline phosphatase in serum and saliva has been proved to be the practical and reliable candidate markers with which to assess bone turnover^36^. In a study, acid, tartrate-resistant acid, and alkaline phosphatase changes in serum and alveolar bone during an orthodontic tooth movement cycle were examined in 288 adult male Sprague–Dawley strain rats^37^. It was reported that in the pooled treatment data, a peak value of alkaline phosphatase occurred at day 7 in serum and bone with a significant drop at day 10 (p < 0.001). As a result of the same study, it was concluded that both serum and bone phosphatase data clearly support the previous histomorphometric observations of the bone turnover characterized by activation, resorption and formation periods during orthodontic tooth movement^37^. Therefore, in the present study, serum BALP levels were analysed in serum samples taken from the subjects on the 7th day. The mean BALP levels of U2, F1, and F2 groups were increased from T1 to T3, and these changes were statistically significant within groups. The mean BALP level of the control group was almost unchanged. However, the mean value of the U1 group statistically significantly decreased from T1 to T3. This decrease in the BALP level may have been due to the decrease in energy level as ESW passes through the surrounding tissues. In a study using human bone marrow cells, the researchers applied 0.16 mJ/mm^2^ of low energy and 250, 500, 1000, 2000, and 3000 impulses with focused ESW applicators, and they reported that there was a significant difference in cell proliferation and the alkaline phosphatase level between the control group and focused ESW group^38^. This and another study suggest that low-dose and low-impulse ESW applications have a biostimulant effect^38,39^. However, it should be noted that cell cultures were used in these studies and the treatment was directly applied to the cells. In our findings, the focused/1000 impulses (F2) group achieved better results. Another view on this subject can be speculated that combined ESW application and orthodontic force have a more complex effect and the biological response varies depending on the number of ESW impulses and the applicator used. This issue is not completely understood but in a study, it was revealed that various growth hormones suppress alkaline phosphatase during the proliferation and differentiation of osteoblasts^40^.

Stereological analysis allows evaluation of two dimensional cross-sections as three dimensional. So it accepted as a superior method in histological analysis methods^20^. In the present study, Cavalier method which is an effective and easy method was used. According to the stereological results of this study, the amount of new bone, connective tissue and capillary volumes were higher in almost all study groups compared to the control group. The analysis of the results indicated that the mean values of these three measurements in the F1 and F2 groups are significantly higher in comparison to control group. These stereological data were supported with increased OTM in ESW groups, particularly in F2 group. The fact that there were significant increases in the formation of new bone, connective tissue and capillary volumes shows that the combined use of orthodontic force and ESW led to significant increases in bone formation in parallel with the increase in OTM.

The medical field has already acknowledged bio stimulant effect of the ESW treatment^12,13,29,33,34^. It was reported that ESW application enables the differentiation of bone marrow stromal cells into osteoprogenitor cells, increases the release of various growth factors and triggers new vascularization^33,34^. In a study it has been proposed to accelerate the formation of new blood vessels if 0.12 mJ/mm^2^-500 impulses low energy shock waves were applied to the Achilles tendon—bone junction^36^. The results of the present study are in line with this and other studies^10,16,29,33,34,36^. As a result of the stereological volume evaluations in the present study, it was revealed that there was a close relationship between new capillary formation and bone deposition.

In the present study, the side effects of weekly ESW application on vital organs, such as eyes and brain, and cells were not investigated. Further studies are required in this area to the determine the optimum dose, frequency, application method of ESW. Quality experimental studies are needed to prevent the potential side effects that can be caused by using ESW during orthodontic treatment.

## Conclusions

- Once a week for three weeks application of shock-wave treatment of focused 1000 impulses at energy flux density 0.19 mj/mm^2^, with a pulse rate of 5 pulses per second caused significant increases in the amount of orthodontic tooth movement.
- Once a week for three weeks application of shock-wave treatment of focused 500 or 1000 impulses caused significantly high new bone, connective tissue, and capillary formation.
- For orthodontic tooth movement acceleration, the formation of new bone and connective tissue, the focused shock-wave application was more effective than unfocussed.

## Abbreviations

OTM: Orthodontic tooth movement
ESW: Extracorporeal shock wave(s)
F1: Focused/500 impulses extracorporeal shock wave applied group
F2: Focused/1000 impulses extracorporeal shock wave applied group
U1: Unfocused/500 impulses extracorporeal shock wave applied group
U2: Unfocused/1000 impulses extracorporeal shock wave applied group
C: Control group
T0: Day 0
T1: Day 7
T2: Day 14
T3: Day 21
BALP: Bone-specific alkaline phosphatase
ELISA: Enzyme-linked immunosorbent assay
CE: Coefficient of error
CV: Coefficient of variation
ANOVA: Analysis of variance
SVE: Stereological volume estimation
